# P-1033. Post-transplant Outcomes in Kidney Transplant Recipients with Invasive Fungal Infections Prior vs. After the Emergence of SARS-CoV2

**DOI:** 10.1093/ofid/ofae631.1223

**Published:** 2025-01-29

**Authors:** Jiashu Xue, Mary Grace Bowring, Darin B Ostrander, Nitipong Permpalung, Lucy X Li

**Affiliations:** Johns Hopkins University, Baltimore, Maryland; Johns Hopkins University, Baltimore, Maryland; Johns Hopkins University, Baltimore, Maryland; Johns Hopkins University School of Medicine, Baltimore, Maryland; Johns Hopkins University, Baltimore, Maryland

## Abstract

**Background:**

Invasive fungal infections (IFIs) are a serious complication in kidney transplant recipients (KTRs), leading to increased mortality and graft failure. Given changes in care associated with pandemic conditions and potential compounding effects of COVID-19 infection, we sought to determine whether the emergence of SARS-CoV2 contributed to a rise in adverse transplant outcomes associated with IFIs.

**Figure:**
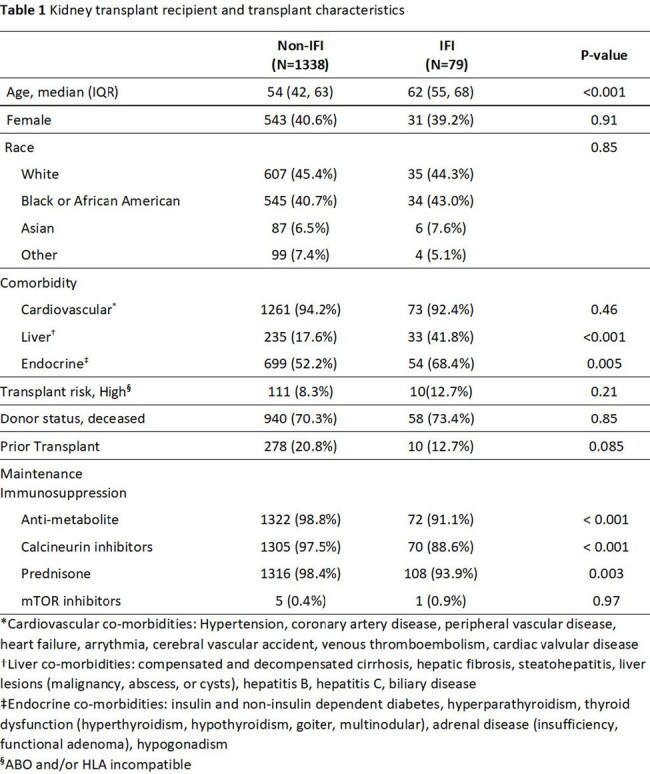

**Methods:**

We performed a retrospective study of adult KTRs transplanted at Johns Hopkins from 2012-2018 with follow up through 5/2023. IFI diagnoses were based on EORTC/MSGERC criteria. If a patient had more than one IFI, the date of first IFI served as the index exposure. KTRs with and without IFIs were matched 1:1 on time-post-KT. We followed patients until all-cause graft loss (ACGL), a composite outcome of graft failure and mortality, and estimated cumulative incidence of ACGL prior vs. after emergence of SARS-CoV2 in 2/2020. We then determined whether the association between IFI and ACGL varied before vs during SARS-CoV2 pandemic using Cox regression with an interaction term and adjusting for age, transplant risk, and cardiovascular disease.

**Table 2.**
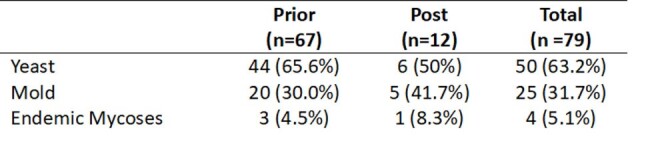
First post-transplant invasive fungal infection among kidney transplant recipients prior vs. post emergence of SARS-CoV2

**Results:**

Among 1453 KTRs, 79 (5.4%) had proven/probable IFIs of which 12 occurred after emergence of SARS-CoV2 (Table 1). Yeast caused the majority of IFIs (50/79) over the study period, but mold (5/12) and yeast (6/12) were equally represented among IFIs occurring after the emergence of SARS-CoV2 (Table 2). Prior to the pandemic, ACGL occurred in 66% (44/67) of KTRs with IFIs compared to 31% (414/1338) of KTRs without IFIs (log-rank p < 0.001). During the pandemic, ACGL occurred in 75% (9/12) of KTRs with IFIs compared to 31% of KTRs without IFIs (log-rank p < 0.001) (Fig 1). KTRs with IFIs had a 2-fold greater risk of ACGL (aHR 2.05, 95% CI 1.17-3.57, p = 0.012) prior to the pandemic and a 5.5-fold greater risk of ACGL (aHR 5.54, 95% CI 1.17-26.24, p < 0.01) during the pandemic. The risk of ACGL associated with IFIs was significantly different after the emergence of SARS-CoV2 (p-interaction term < 0.001; Fig 2).

**Figure 1.**
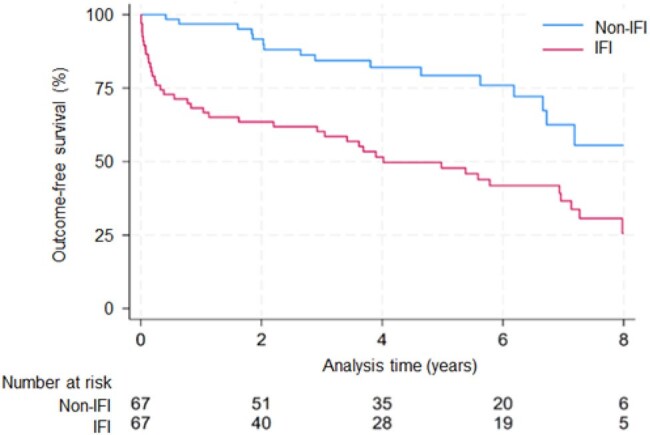
ACGL-free survival of KTR with IFIs vs. without IFIs

**Conclusion:**

KTRs with IFIs had higher rates of ACGL during the pandemic, and molds constituted a greater proportion of the IFIs during the pandemic. The emergence of SARS-CoV2 seems to have shifted the epidemiology of IFIs among KTRs, leading to increased ACGL.

**Figure 2.**
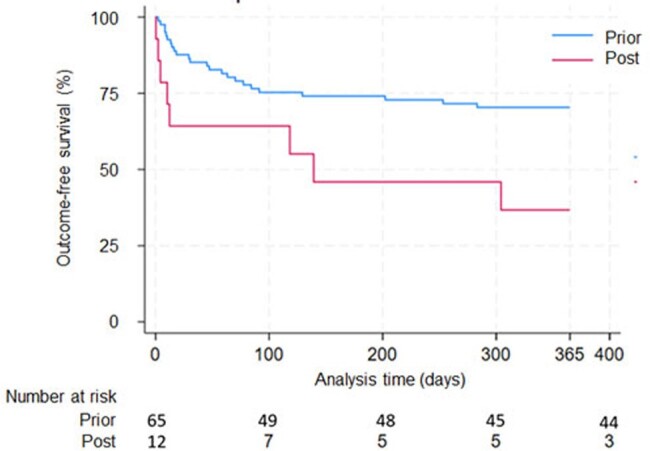
ACGL-free survival of KTR with IFIs prior vs. after emergence of SARS-CoV2

**Disclosures:**

**Nitipong Permpalung, MD, MPH**, CareDx: Grant/Research Support|Cidara Therapeutics: Grant/Research Support|ClearView: Advisor/Consultant|IMMY Diagnostics: Grant/Research Support|Merck: Grant/Research Support|Pearl Diagnostics: Grant/Research Support|Pulmicide: Advisor/Consultant|Scynexis: Grant/Research Support

